# A population-specific low-frequency variant of *SLC22A12* (p.W258*) explains nearby genome-wide association signals for serum uric acid concentrations among Koreans

**DOI:** 10.1371/journal.pone.0231336

**Published:** 2020-04-09

**Authors:** Sun-Wha Im, Jeesoo Chae, Ho-Young Son, Belong Cho, Jong-Il Kim, Jin-Ho Park

**Affiliations:** 1 Genomic Medicine Institute, Medical Research Center, Seoul National University, Seoul, Republic of Korea; 2 Department of Biomedical Science, Seoul National University Graduate School, Seoul, Republic of Korea; 3 Departments of Family Medicine, Seoul National University College of Medicine and Seoul National University Hospital, Seoul, Republic of Korea; 4 Cancer Research Institute, Seoul National University, Seoul, Republic of Korea; Mayo Clinic Arizona, UNITED STATES

## Abstract

Prolonged hyperuricemia is a cause of gout and an independent risk factor for chronic health conditions including diabetes and chronic kidney diseases. Genome-wide association studies (GWASs) for serum uric acid (SUA) concentrations have repeatedly confirmed genetic loci including those encoding uric acid transporters such as *ABCG2* and *SLC9A2*. However, many single nucleotide polymorphisms (SNPs) found in GWASs have been common variants with small effects and unknown functions. In addition, there is still much heritability to be explained. To identify the causative genetic variants for SUA concentrations in Korean subjects, we conducted a GWAS (1902 males) and validation study (2912 males and females) and found four genetic loci reaching genome-wide significance on chromosomes 4 (*ABCG2*) and 11 (*FRMD8*, *EIF1AD* and *SLC22A12-NRXN2*). Three loci on chromosome 11 were distributed within a distance of 1.3 megabases and they were in weak or moderate linkage disequilibrium (LD) states (*r*^*2*^ = 0.02–0.68). In a subsequent association analysis on the GWAS loci of chromosome 11 using closely positioned markers derived from whole genome sequencing data, the most significant variant to be linked with the nearby GWAS signal was rs121907892 (c.774G>A, p.W258*) of the *SLC22A12* gene. This variant, and each of the three GWAS SNPs, were in LD (*r*^*2*^ = 0.06–0.32). The strength of association of SNPs with SUA concentration (negative logarithm of *P*-values) and the genetic distance (*r*^*2*^ of LD) between rs121907892 and the surrounding SNPs showed a quantitative correlation. This variant has been found only in Korean and Japanese subjects and is known to lower the SUA concentration in the general population. Thus, this low-frequency variant, rs121907892, known to regulate SUA concentrations in previous studies, is responsible for the nearby GWAS signals.

## Introduction

Uric acid is the final product of purine metabolism produced from xanthine and hypoxanthine by the action of xanthine oxidase. Uric acid is mainly present as urate under normal physiological conditions. Homeostasis of serum uric acid (SUA) concentration is maintained through a balance between oral intake and renal/intestinal excretion. Prolonged elevation of SUA concentrations—hyperuricemia—accelerates the formation of monosodium urate crystals and causes gout. Hyperuricemia is independently associated with hypertension, diabetes, chronic kidney diseases and cardiovascular mortality [1–3]. In addition, recent clinical or experimental studies have suggested that hyperuria contributes to the development of metabolic syndrome [4].

The genetic contribution to SUA concentration is estimated at 40–70% [5]. To identify underlying genetic alterations involved in the regulation of SUA concentration, genome-wide association studies (GWASs) have been conducted in many populations, and genetic loci covering *ABCG2* and *SLC9A2* have been found most repeatedly and significantly [6–10]. Most of the genes identified in current GWASs seem plausible contributors to SUA concentrations because they encode for proteins that are responsible for uric acid excretion in the kidney or intestines, including the genes *ABCG2* and *SLC9A2* [11]. However, except for rs2231142 in *ABCG2*, most single nucleotide polymorphisms (SNPs) that have been reported to be significant so far are located in intronic or intergenic regions, and causative variants have not been identified for most loci. These collectively explained up to 7% of the inter-individual SUA variance in serum concentration, and the proportion explained by *ABCG2* and *SLC9A2* alone was about 2–3% [6,7,9]. Thus, much heritability is yet to be explained and many genetic variants remain to be identified.

While GWASs to date have provided valuable clues about the genetic background for SUA concentrations, additional GWASs using common variants with low effect are unlikely to be able to explain the residual heritability robustly. This “missing heritability” problem has also been raised in many other GWASs [12]. One hypothesis is that common variants with weak effects might have been found in GWASs because of their linkage disequilibrium (LD) relationships with causative variants with low-frequencies and large effects. To find such low-frequency causative variants responsible for GWAS signals, resequencing of target sites or whole genome sequencing (WGS) is required. Here, we identify such a low-frequency causative variant and its relationship with common variants in GWAS signals.

## Results

### GWAS and validation

**Fig 1:**
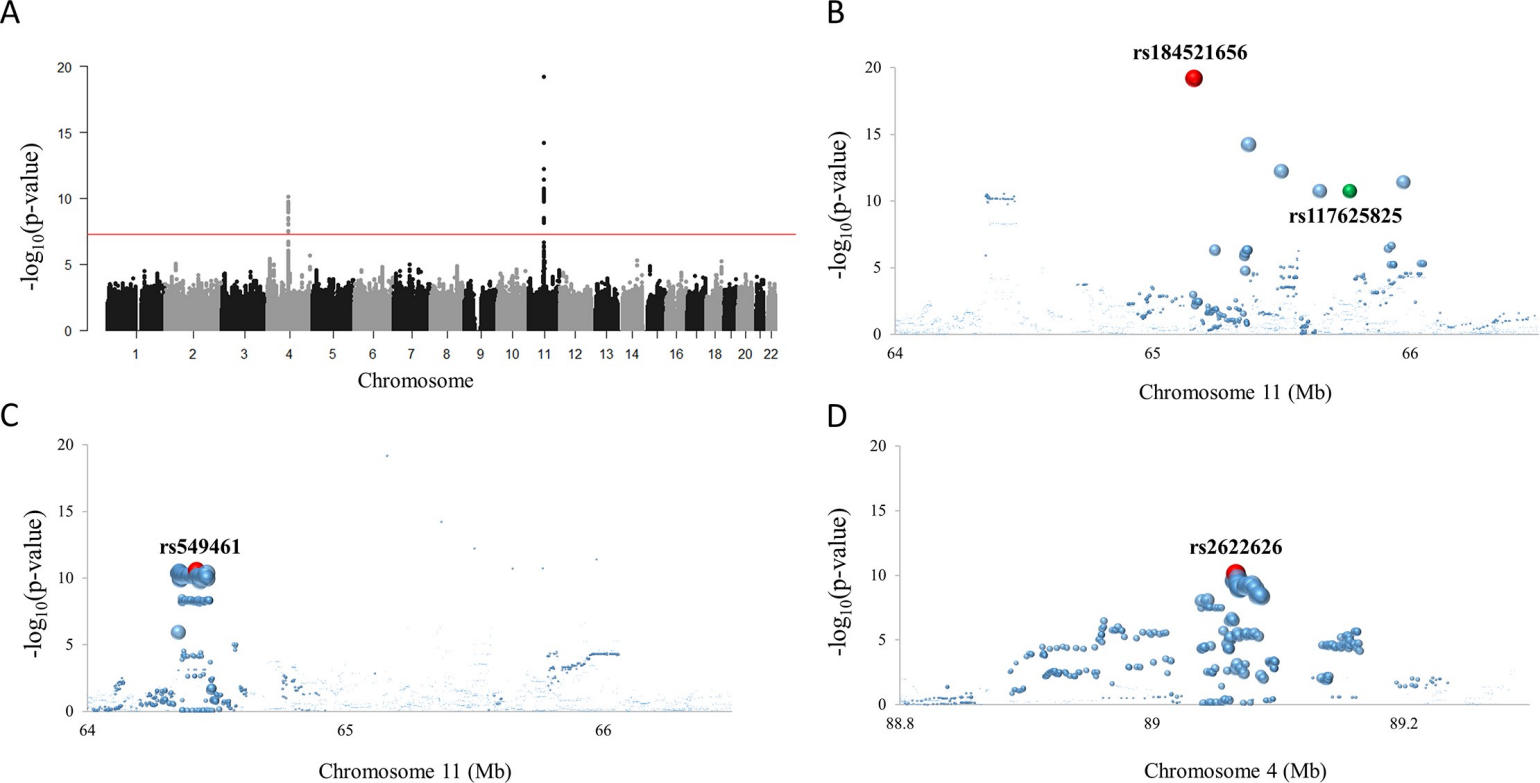
(**A**) Manhattan plot of the GWAS results for SUA concentration. The horizontal red line represents the genome-wide significance level. Regional plots of the GWAS signals are shown for chromosome 11, 65–66 Mb (**B**), 64–65 Mb (**C**) and chromosome 4, 89 Mb (**D**). The red circle indicates the most significant SNP in the locus, and the circle size is proportional to the strength of LD (*r*^*2*^) with the most significant SNP. (**B**) Rs184521656 and rs117625825 are in a moderate LD relationship (*r*^*2*^ = 0.68).

The baseline characteristics for study participants are described in S1 Table. The median SUA concentration was 6.4 mg/dL. As shown in the quantile–quantile (Q–Q) probability plot, the observed *P*-values of the GWAS were well fitted to expected values (S1 Fig). The genomic inflation factor was calculated as 1.0, demonstrating the homogeneity of the study population. The four association signals were found to reach genome-wide significance level (*P* < 5.0E^–8^; Table 1 and Fig 1). The most significant genetic association was found on chromosome 11, in a 65 Mb region (Fig 1B). The six SNPs with a low minor allele frequency (MAF) of 1.2–1.6% and *p* < 1E^–10^ were widely distributed over the region (Fig 1B). Because rs184521656 and the other five SNPs were in a moderate state of LD (*r*^2^ = 0.66–0.77) and those SNPs excluding rs184521656 showed strong LD relationships with each other (*r*^2^ > 0.95), these adjacent SNPs were considered as potentially separate signals (S2 Table). Rs184521656 is located on the intronic region of *FRMD8*. The other five SNPs are located on noncoding or intergenic regions of different genes and the areas in which they were located contain many genes. The third strongest signal was from chromosome 11, in a 64 Mb region. Rs549461 (*P* = 2.8E^–11^, MAF = 22%) and many significant SNPs with LD relationships (*r*^2^ > 0.83) are located on the *SLC22A12-NTXN2* region (Fig 1C). Another signal (rs2622626, *p* = 7.5E^–11^, MAF = 30%) was located on the *ABCG2* region of chromosome 4 (Fig 1D).

**Table 1:** Results of the genome-wide association and validation studies for serum uric acid concentrations.

SNP	Chr	Position (bp)	Nearest genes	Minor allele	Major allele	GWAS (*n* = 1902)			Validation study (*n* = 2912)			Combined (*n* = 4814)
						MAF (%)	Beta (SE)	*P*	MAF (%)	Beta (SE)	*p*	*p*
rs184521656	11	65161450	*FRMD8* (intron)	T	C	1.2	–1.70 (0.18)	6.5E^–20^	1.3	–0.99 (0.14)	6.0E^–12^	1.1E^–28^
rs117625825	11	65765725	*EIF1AD* (3′ UTR)	A	G	1.6	–1.11 (0.16)	1.9E^–11^	1.5	–0.84 (0.13)	1.2E^–10^	1.9E^–20^
rs549461	11	64423032	*SLC22A12-NRXN2* (intron)	A	G	22.4	–0.33 (0.05)	2.8E^–11^				
rs2622626	4	89066715	*ABCG2 (*intron)	C	A	30.0	–0.30 (0.05)	7.5E^–11^				

GWAS, genome-wide association study; SNP, single nucleotide polymorphism; bp, base pair; Chr, chromosome; MAF, minor allele frequency; SE, standard error; UTR untranslated region

Because the latter two loci in *SLC22A12-NRXN2* and *ABCG2* were found repeatedly in previous studies [11], we only validated the SNPs on the 65 Mb region of chromosome 11 by increasing the number of individuals sampled. Rs117625825 was chosen as the tag SNP, the best representative of the remaining five SNPs identified using Haploview [13] (S3 Table). Rs184521656 and rs117625825 were genotyped and analyzed as an independent group (Table 1); they were validated and had statistical significance. Because only males were included in the GWAS stage and both males and females were included in the validation study stage, we further tested the effect of these two SNPs (rs184521656 and rs117625825) on the SUA concentration of each male and female subject. In both males and females, these two SNPs were significantly associated with SUA concentration and the direction of their effect was also the same (S4 Table).

### Association study using WGS data

**Fig 2:**
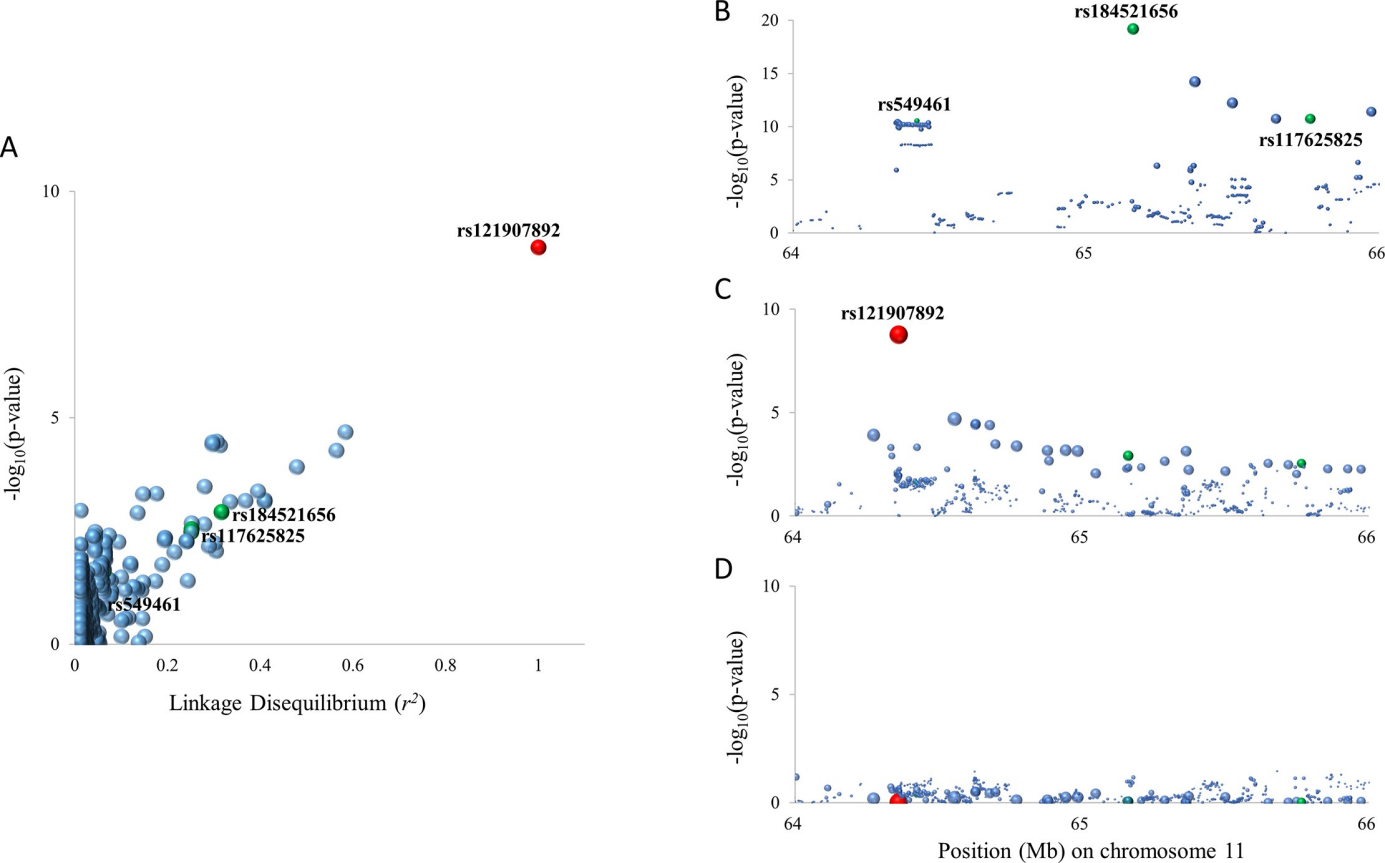
The role of rs121907892 in the GWAS signal on chromosome 11: 64–66 Mb region. All dots in the figure are SNPs showing a genetic association with rs121907892, with *r*^*2*^ values > 0.01. Green circles are representative SNPs of each locus that reached genome-wide significance in the GWAS. The circle size is proportional to the strength of LD (*r*^*2*^) with rs121907892 (red circle). (**A**) Correlation between LD value (*r*^*2*^) with rs121907892 and the significance of association (–log_10_ *p*) of SNPs with serum uric acid (SUA) concentrations. (**B**) Regional plot for the results of the GWAS for SUA concentration. (**C**, **D**) Regional plots for the results of the association analysis for SUA concentration using SNPs derived from whole genome sequencing data before (**C**) and after (**D**) the conditional analysis incorporating rs121907892.

**Fig 3:**
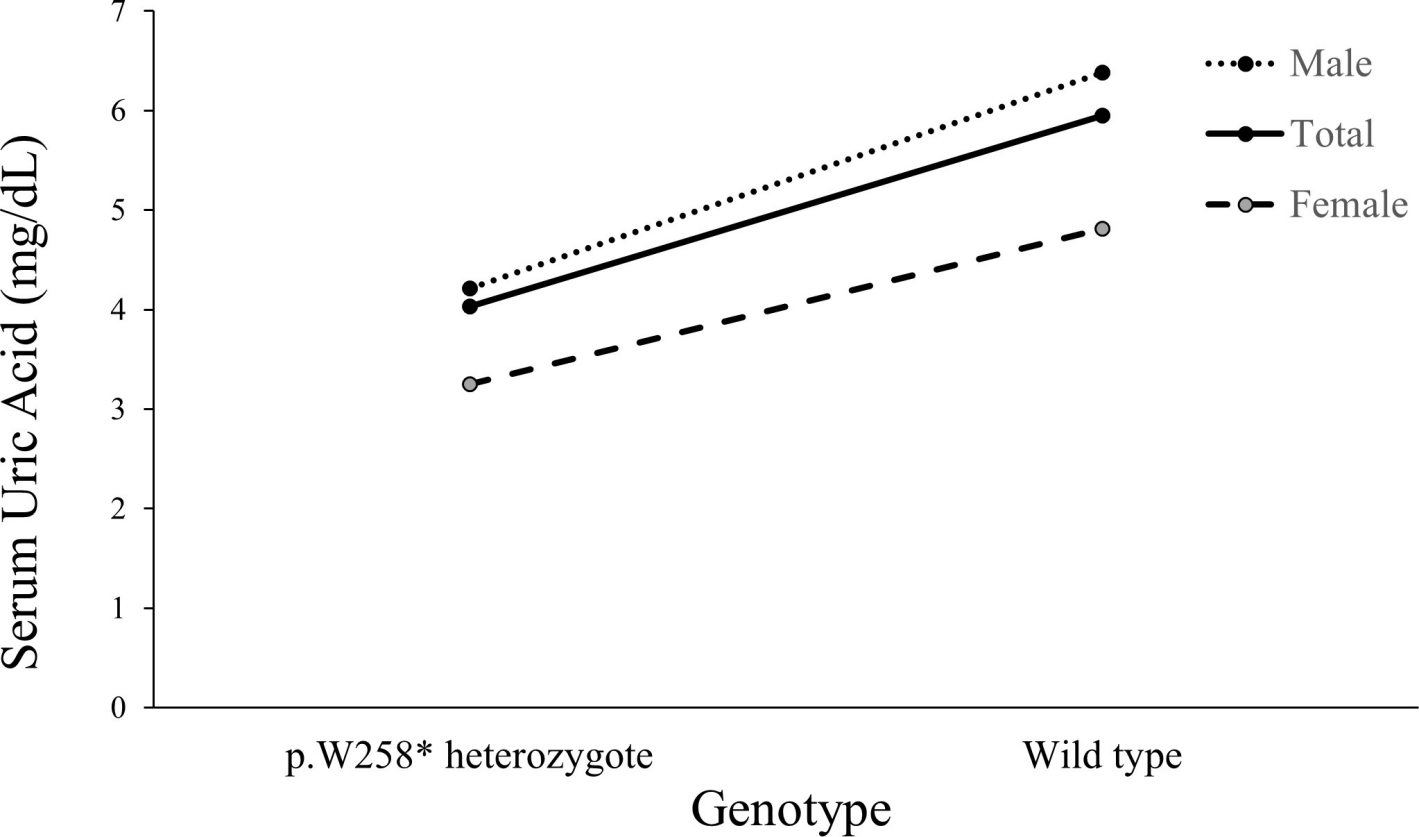
Effect of the p.W258* genotype on serum uric acid concentration in this study group.

Although rs184521656 and rs117625825 have been validated successfully with statistical significance, it is difficult to regard these SNPs as causative variants because they are located in noncoding regions and the functions of their adjacent genes are insufficient to explain a causal relationship with SUA concentration. To identify more influential variants, we performed additional association analysis using closely spaced markers around the GWAS signal on chromosome 11, 63–67 Mb in extent, extracted from the 797 WGS data that formed part of the validation samples (S2 Fig). The most significant SNP, rs121907892 (*P* = 1.7E^–09^, MAF = 1.2%; Table 2), was positioned at the third nucleotide of amino acid codon #258 of *SLC22A12* (NM_144585). The nucleotide change G>A of this SNP turns p.W258 into a stop codon (p.W258*). Interestingly, the degree of LD (estimated as *r*^2^) between rs121907892 and surrounding SNPs and the negative logarithm of the *P*-value (–log_10_ *p*) of their association showed positive correlation, which was also observed in the relationship with rs184521656, rs117625825 and rs549461 (Fig 2A). When associations of the SNPs with SUA concentration were reanalyzed after adjusting for rs121907892, their associations were weakened greatly (Fig 2B–2D and S5 Table). To confirm the association between rs121907892 and SUA concentration in larger samples, rs121907892 was genotyped and analyzed in an entire sample including discovery (GWAS) and validation samples. Among a total of 4708 individuals that were successfully genotyped, 130 had a heterozygous p.W258* variant and none had a homozygous variant (MAF = 1.4%). The strongest association was observed (*P* = 2.3E^–88^) and the SNP explained 8.1% of the inter-individual variance of SUA concentration in our study population (Table 2). The mean SUA concentrations of those with or without p.W258* were 4.03 and 5.95 mg/dL, respectively (Fig 3 and S6 Table).

**Table 2:** The association of rs121907892 with SUA concentration.

SNP	Chr	Position (bp)	Nearest gene	Minor allele	Major allele	WGS (*n* = 797)			Validation study (*n* = 4708)		
						MAF (%)	Beta (SE)	*p*	MAF (%)	Beta (SE)	*p*
rs121907892	11	64361219	*SLC22A12* (p.W258*)	A	G	1.2	–1.68 (0.28)	1.7E^–09^	1.4	–2.10 (0.10)	2.3E^–88^

Chr, chromosome; WGS, whole genome sequencing; MAF, minor allele frequency; SE, standard error

## Discussion

Using a stepwise approach, we found that a low-frequency SNP, rs121907892, was the lead variant of nearby GWAS signals for SUA concentrations. First, in our GWAS, we found four association signals reaching genome-wide significance, two previously established common SNPs (rs2622626 and rs549461) and two new low-frequency SNPs (rs184521656 and rs117625825). Although the latter two were validated using a subsequent study with increased sample size, because of their location in a noncoding region and weak cogency of their surrounding genes, we anticipated that there might be a causative variant nearby, and conducted further association analysis using more closely spaced markers derived from WGS data. The SNP rs121907892—the most significant variant found in this analysis—shows a LD relationship with surrounding variants including the three SNPs that reached genome-wide significance in the initial GWAS in chromosome 11 at 64–65 Mb (rs184521656, rs117625825 and rs549461). The degrees of LD between rs121907892 and each surrounding SNP and the significance of their associations with SUA concentrations were closely related. In addition, in the conditional analysis on rs121907892, the associations of these SNPs with SUA concentrations disappeared.

The *SLC22A12* gene encodes a urate transporter in renal proximal tubules serving to reabsorb urate [14]. A *SLC22A12* p.W258* variant was first found in a Japanese patient with idiopathic renal hypouricemia and exercise-induced acute renal failure [14], and has been reported further in Korean and Japanese patients with hereditary renal hypouricemia [15,16]. In addition to patients with this Mendelian inherited disorder, the effects of p.W258* on SUA concentrations or renal functions have been studied in the general population. The Suita study targeted a community-based general population in Japan and sequenced the *SLC22A12* gene of 24 subjects with low SUA concentrations; it found that those with a homozygous or heterozygous p.W258* variant had low SUA concentrations but normal creatinine concentrations [17]. They suggested that loss-of-function variants of *SLC22A12* might not be harmful in the general population. Another study examined the genotype distribution of p.W258* and SUA concentrations in 5023 Japanese subjects from the general population [18]. Five individuals with a homozygous p.W258* variant had SUA concentrations of < 1 mg/dL, and those with a heterozygous p.W258* variant had a significantly lower mean SUA concentration than did subjects with a wild-type allele, although they had a wider range of SUA concentrations (0.8–7.8 mg/dL) than did subjects with a homozygous p.W258* variant. The *SLC22A12* p.W258* variant has been found only in Japanese and Koreans (http://www.1000genomes.org). Its allele frequency in Japanese and Korean general populations has been reported to be 0.02 and 0.01, respectively [18,19], consistent with our results. In our study, 130 subjects with the heterozygous p.W258* variant had slightly higher estimated glomerular filtration rate values than those without the variant (90.6 vs 88.1 mL/min/1.73 m^2^), but this was not statistically significant [20]. Thus, the role of *SLC22A12* p.W258* in lowering the SUA concentration is relatively clear, but it does not seem to have adverse effects on renal function in the Japanese and Korean general populations.

Targeted sequencing of the *SLC22A12* gene has identified low-frequency variants greatly affecting SUA concentrations in the general population. In addition to p.W258*, Iwai *et al*. identified additional rare variants (MAF < 0.01) in subjects with low SUA concentrations. Among these, p.R90H and p.R477H showed statistically significant associations with hypouricemia; a deletion of p.D313–P333 was identified in only one subject, but it was proved experimentally that it abolished the urate transport activity of SLC22A12 [17]. In addition, a subject with the p.R228E variant had a low SUA concentration, although this missense variant was not proved experimentally and was not statistically significant because it was found in only one subject. Direct sequencing of exons 7 and 9 of the *SLC22A12* gene revealed a deletion (p.L415_G417del) and a missense variant (p.T467M) in 1.87 and 5.56% of the Czech and Slovak Roma populations, respectively [21]. Although that study did not directly address the association of these variants with SUA concentration, these variants were initially found in patients with primary renal hypouricemia in the same geographic area. We reviewed rare variants of the *SLC22A12* gene in our WGS data and found three missense variants (p.R90H, p.R447H and p.Q382L) and one splicing site variant (c.661+1G>A). No subject had a homozygotic or a compound heterozygotic form of these variants; instead, all variant-positive subjects had only one of them in a heterozygous form and also showed low SUA concentrations (S7 Table). The p.Q382L variant was reported in a Japanese patient with renal hypouricemia as a compound heterozygotic form of p.W258*/p.Q382L [16]. The splicing site variant (c.661+1G>A) was found in only one person in our study. This was submitted to the ClinVar database (https://www.ncbi.nlm.nih.gov/clinvar/), and was classified as “Uncertain significance” under the “Familial renal hypouricemia” condition (accessed 31 July 2019) [22].

Population genetic models have demonstrated that a variant’s allele frequency is inversely related to the magnitude of its effect on a phenotype [23]. According to this, the common variants with MAF ≥ 0.05 that are mostly used in GWASs have inherently low “regression effects.” Therefore, there have been emerging attempts to find more powerful low-frequency variants. These variants inevitably show a population-specific distribution, because they are recent [24]. In addition to the 1000 genome projects involving 26 populations from all over the world, efforts are under way to create reference panels using WGS approaches in particular sample sets including the British (UK10K), Dutch (Genome of the Netherlands) [25], Sardinian [26] and Icelandic (deCODE Genetics) [27] populations. These have shown that population-based panels are particularly effective in discovering genotypes with a low population frequency. In these studies, some low-frequency variants strongly associated with specific phenotypes have been identified, as summarized recently [28]. The phenotypes included various complex traits or diseases such as blood lipid or glucose concentrations, bone mineral density, height, type 2 diabetes, and Alzheimer’s disease, but to our knowledge there have been no results for SUA concentrations.

The role of the common noncoding variant found in GWASs has been revealed through subsequent studies in some cases. Experiments have shown that T>C base changes in rs1421085, an intronic variant of the *FTO* gene found in GWASs on obesity, is involved in the regulation of adipocyte thermogenesis by increasing the expressions of nearby *IRX3* and *IRX5*, but not *FTO* [29]. Rs7085104 upstream of *AS3MT* gene, a top GWAS-identified SNP associated with schizophrenia, is linked with a variable number tandem repeat sequence in exon 1 of *AS3MT* that is involved in the expression of the arsenite methyltransferase isoform (AS_3_MT^d2d3^) [30]. Surakka *et al*. showed that low-frequency functional variants that cause Mendelian-inherited dyslipidemia or have a known impact on lipids have larger average effects than the common variants found in GWASs, and contribute considerably to population variance in blood lipid concentrations [31]. Some of these low-frequency variants explain the association of common variants in the overlapping loci [31]. In our study, rs121907892 explained the association of surrounding common or low-frequency variants located over 1 Mb and at—least in Koreans—it might explain the signals found at this locus in previous GWASs for SUA concentration. This study linked the previously found GWAS signals and a variant that causes Mendelian-inherited disorders. It is also clearly shown that the degree of associations of SNPs with the phenotype correlates with the degree of LD between the low-frequency lead SNP and its surrounding SNPs.

The biggest weakness of this study is that rs121907892 is found only in Koreans and Japanese, so the results cannot be applied to other populations. Because this genetic locus has been repeatedly found in GWASs of other populations, there might be other low-frequency variants such as rs121907892. However, this study was conducted only in Koreans, so we could not find such variations in other ethnic groups. In addition, for many GWAS results for common variants lacking known molecular mechanisms, it will be necessary to use multifaceted approaches such as deep sequencing or functional experiments to discover their mechanism of action or the actual functioning variants surrounding them.

## Methods

### Study design and baseline measurements

The design of this study has been described previously [32]. A schema of this study is shown in S2 Fig. The subjects enrolled for this GWAS included unrelated adult male Koreans aged 20–65 years who visited the Seoul National University Hospital (SNUH, Seoul, Republic of Korea) for regular health checkups from May 2011 to December 2013. This study was originally designed to investigate the genetic background of abdominal obesity and metabolic syndrome and only male participants were recruited during that period. Participants with conditions that might influence body weight were excluded: individuals who had 1) been diagnosed with thyroid diseases or taken thyroid medication; 2) taken medication or undergone a procedure or operative treatment for obesity within 3 months of enrollment; 3) a medical history of stroke, cardiovascular diseases, diabetes mellitus treated with medication, cancer, or abdominal surgery. Repeated examinees and those with insufficient blood for testing were also excluded. In addition, individuals taking medications that could influence the SUA concentration, including allopurinol and antituberculotics, were excluded. After application of the exclusion criteria, 1,947 males were recruited for the GWAS. Since 2014, it has expanded to include both male and female participants. In this phase, 2,912 individuals (1,557 males and 1,355 females) were enrolled for the validation study. This study was approved by the institutional review board of SNUH (#C-1701-131-828) and all subjects gave written informed consent.

Anthropometric values including height and weight were measured by trained medical personnel, and the body mass index (BMI, in kg/cm^2^) was calculated subsequently. Blood tests were conducted after ≥ 12 h of fasting and the SUA concentration was measured by enzymatic colorimetric method using a COBAS 8000 c 702 analyzer (Roche Diagnostics, Japan) in mg/dL. Participants filled out a standardized questionnaire routinely used for health checkups that included items regarding previous diseases or medication history.

### GWAS

Genome-wide SNP genotyping and imputation procedures have been described previously [32]. The Illumina HumanCore BeadChip kit (Illumina, San Diego, CA, USA) was used for genome-wide SNP genotyping of 1,947 subjects. Individuals with call rate < 0.88 were excluded. After SNPs with Hardy–Weinberg equilibrium (HWE) with *P* < 1.0E^–6^, call rate < 95%, or MAF < 1% had been excluded, the remaining 264,283 SNPs were used for genotype imputation. For the imputation, 1000 Genomes ASN Phase I integrated variant set release (v. 3) in NCBI build 37 (hg19) was used as the reference panel. We used PLINK (v. 1.07) for strand alignment, SHAPEIT2 for phasing, and IMPUTE2 for imputation [33–35]. Of the imputed SNPs, only those variants with Info Score ≥ 0.9 were selected. A total of 4,414,664 SNPs that were either genotyped or imputed with high confidence were used for the GWAS. One member of a pair of relatives was randomly selected based on genome-wide identity-by-descent estimates implemented in PLINK and the other member was excluded. In addition, after excluding those for whom phenotype information was not available, 1,902 males with both genotype and phenotype information were included in the GWAS. The distribution of SUA concentrations is described in S3 Fig. Genetic associations of SUA concentrations were tested using PLINK using a linear model adjusted for age and BMI. R software (v. 3.4.1) was used for drawing the SUA concentration distribution, Q–Q and Manhattan plots (https://www.R-project.org/). Integrative Genomics Viewer software (v2.3.6) was used for review of the association results [36]. SNPs were annotated to obtain the information including amino acid changes or population frequencies using ANNOVAR [37].

### Validation study and meta-analysis

A validation study was conducted by increasing the number of subjects on two SNPs (rs184521656 and rs117625825) that were found to be the most significant in our GWAS (S2 Fig). Of the 2,912 subjects who were included for the validation study, 2,691 and 2,699 individuals were genotyped successfully using the Taqman method for rs184521656 and rs117625825, respectively. Detailed information about the probes used for Taqman genotyping is listed in S8 Table. ViiA 7 real-time quantitative polymerase chain reaction equipment and embedded software were used (Thermo Fisher Scientific, Waltham, MA, USA).

Association analysis between SUA concentration and these two SNPs were performed in the same method as GWAS using a linear model implemented in PLINK. Because the validation study included both male and female subjects, gender was added as another covariate in addition to age and BMI. The results of the GWAS and validation study for these two SNPs were combined to obtain an overall estimate of *P*-value through meta-analysis using inverse variance-based analysis assuming a fixed-effect model implemented in PLINK (Table 1).

### Association analysis of target regions in chromosome 11 using WGS data

Of the 2,912 individuals included in the validation study, 850 were subjected to WGS with a mean depth of 15–30×. Paired-end sequencing reads generated on the Hiseq platform were aligned to the human reference sequence (hg19) and subsequent data processing including making BAM files and genetic variant call format files were performed using the Dynamic Read Analysis for GENomics (DRAGEN) platform by a commercial company (Macrogen Inc., Seoul, S. Korea). SNPs with a calling rate > 0.9 and HWE *P* > 1.0E^–3^ were selected for variants with local read depth ≥ 5 and genotype quality ≥ 20. Samples with heterozygous/homozygous ratios < 2 and call rate > 0.9 were selected; a total of 802/850 subjects passed the sample quality control (QC). Of these, 797 had phenotype data and were included in the final association analysis using WGS data. Of the 32,613 SNPs that passed the QC on the 63–67 Mb region of chromosome 11, only 6,773 SNPs with MAF > 1% were included for the association analysis. Associations of these SNPs with SUA concentrations were evaluated in the same way using PLINK.

### Statistical significance

Associations with *P* < 5.0E^–8^ were considered genome-wide significant in the discovery GWAS stage [6,38]. In the following analysis, associations were considered significant if the *P*-value was lower than the Bonferroni correction level (0.05/the number of tests).

## Supporting information

S1 Fig Quantile–quantile (Q–Q) plot of the genome-wide association study (GWAS). The genomic inflation factor was calculated as 1.
(PDF)

S2 Fig Schema of the study. The numbers in the circles refer to the order in which the study was conducted.
(PDF)

S3 Fig Distribution of serum uric acid (SUA) concentrations in 1902 subjects used for the GWAS. (PDF)

S1 Table The demographics of the study participants. (PDF)

S2 Table Linkage disequilibrium (LD) state (*r*^*2*^) between single nucleotide polymorphisms (SNPs reaching a genome-wide significance on chromosome 11 65 Mb). (PDF)

S3 Table Selection of the tag SNPs. (PDF)

S4 Table The association of rs184521656 and rs117625825 with serum uric acid concentration in males and females. (PDF)

S5 Table Association analysis of rs184521656, rs117625825 and rs549461 using whole genome sequencing (WGS) data before and after adjustment for rs121907892. (PDF)

S6 Table Serum uric acid concentrations according to the rs121907892 genotype in the study population. (PDF)

S7 Table Serum uric acid concentration of subjects with low-frequency variants of *SLC22A12*. (PDF)

S8 Table Probes used for Taqman genotyping. (PDF)
