# Supplementary material for: A population-specific low-frequency variant of SLC22A12 (p.W258*) explains nearby genome-wide association signals for serum uric acid concentrations among Koreans
Source: PLoS One. 2020 Apr 9;15(4):e0231336. doi: 10.1371/journal.pone.0231336 (PMC7145145; doi:10.1371/journal.pone.0231336)

S1 Fig. Quantile–quantile (Q–Q) plot of the genome-wide association study (GWAS). The genomic inflation factor was calculated as 1.

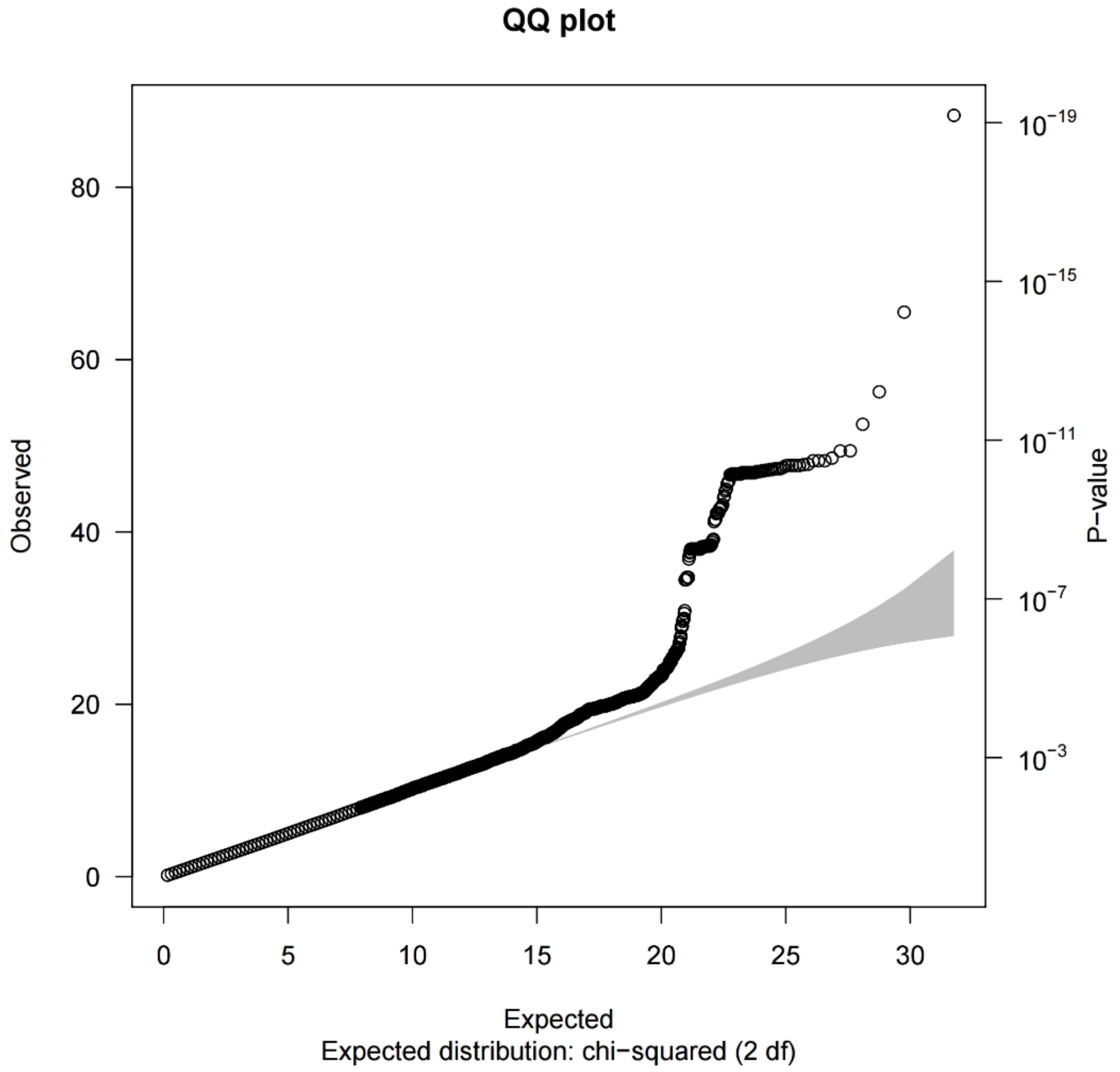

Supplement: S1 Fig — The genomic inflation factor was calculated as 1. (PDF) [file pone.0231336.s001.pdf]
