# Supplementary material for: A population-specific low-frequency variant of SLC22A12 (p.W258*) explains nearby genome-wide association signals for serum uric acid concentrations among Koreans
Source: PLoS One. 2020 Apr 9;15(4):e0231336. doi: 10.1371/journal.pone.0231336 (PMC7145145; doi:10.1371/journal.pone.0231336)

S2 Fig. Schema of the study. The numbers in the circles refer to the order in which the study was conducted.

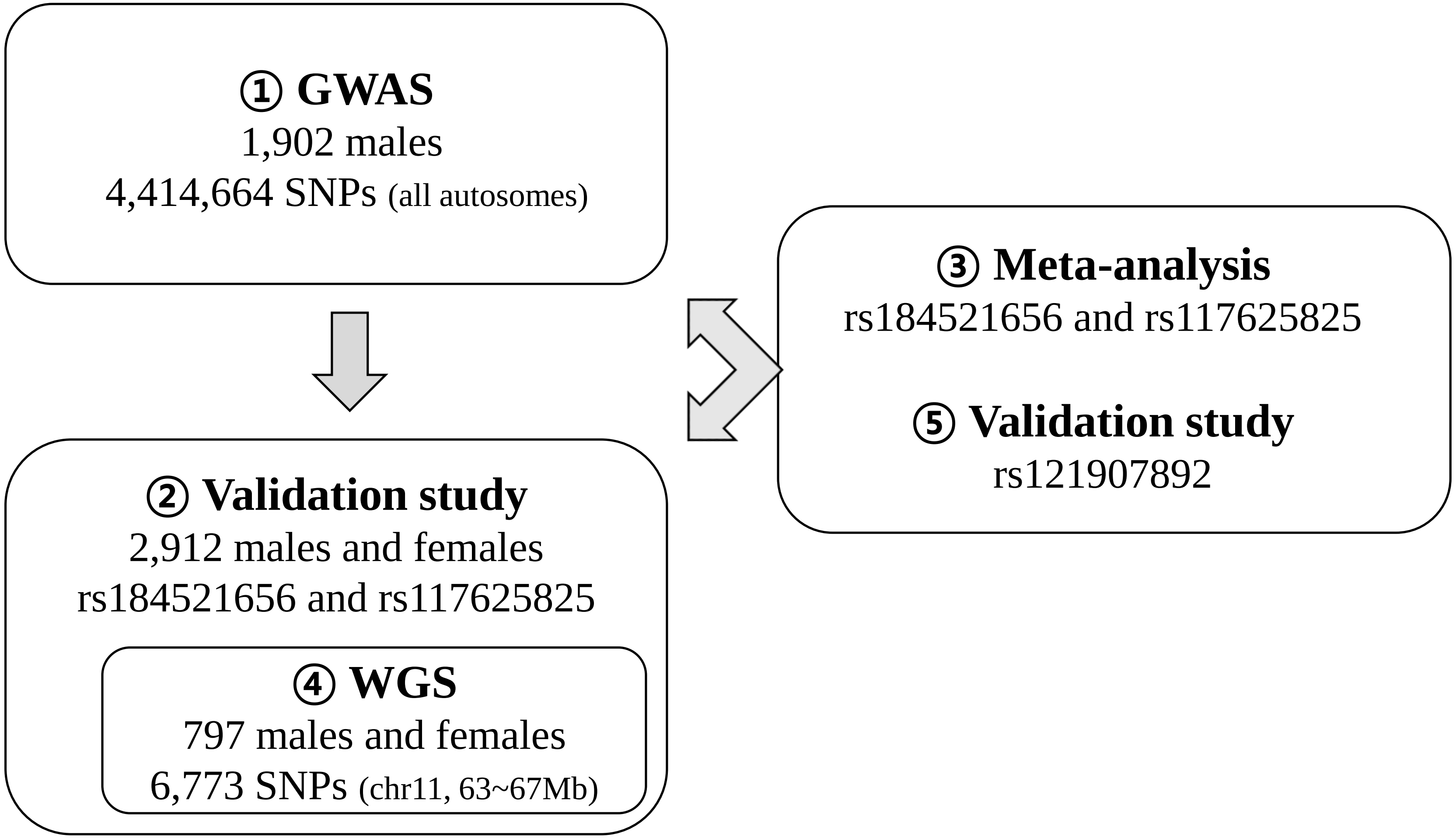

Supplement: S2 Fig — The numbers in the circles refer to the order in which the study was conducted. (PDF) [file pone.0231336.s002.pdf]
