# Supplementary figures and images for: A population-specific low-frequency variant of SLC22A12 (p.W258*) explains nearby genome-wide association signals for serum uric acid concentrations among Koreans
Source: PLoS One. 2020 Apr 9;15(4):e0231336. doi: 10.1371/journal.pone.0231336 (PMC7145145; doi:10.1371/journal.pone.0231336)

**S3 Fig. Distribution of serum uric acid (SUA) concentrations in 1902 subjects used for the GWAS.**

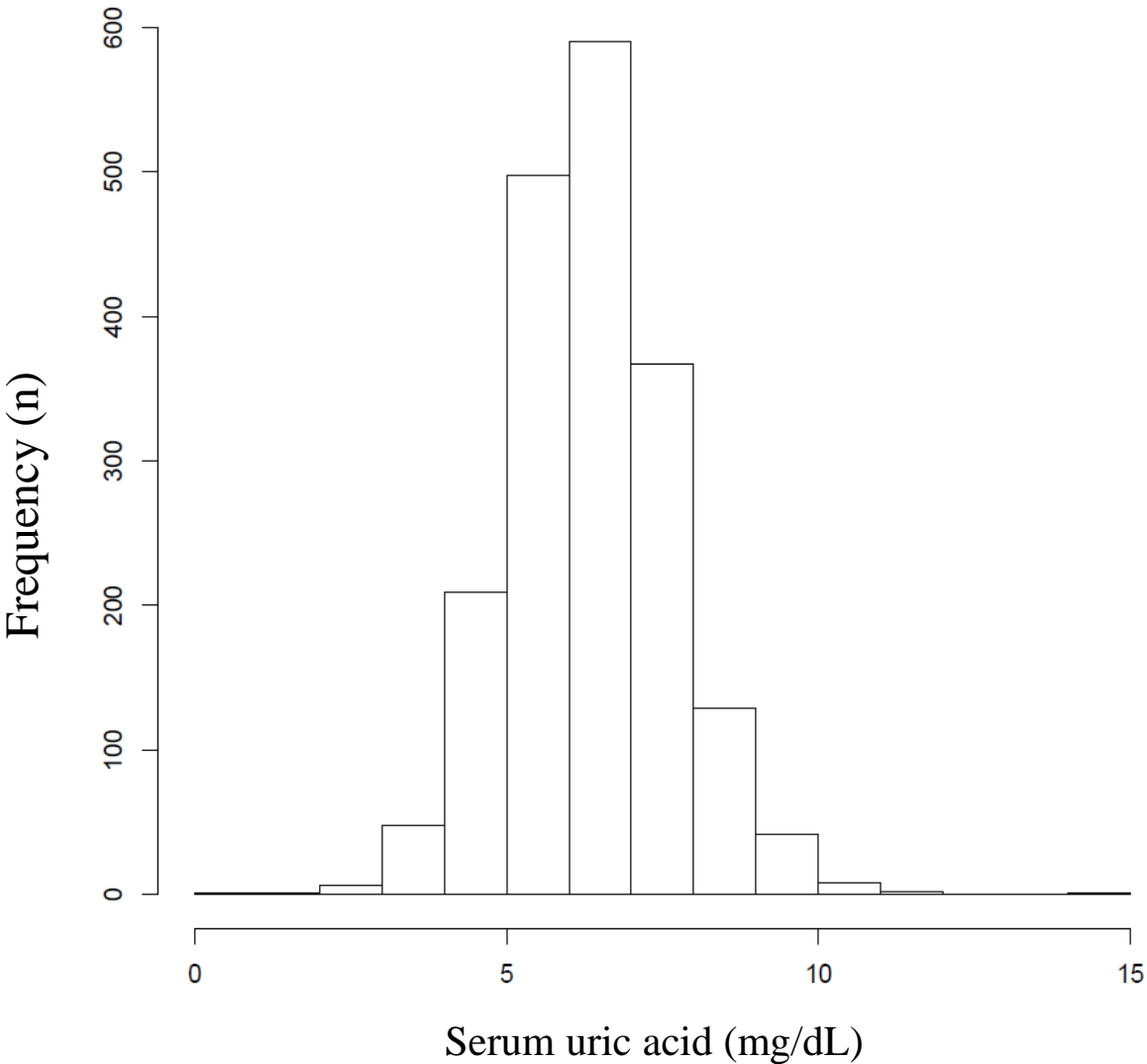

Supplement: S3 Fig — (PDF) [file pone.0231336.s003.pdf]
