# Supplementary material for: A population-specific low-frequency variant of SLC22A12 (p.W258*) explains nearby genome-wide association signals for serum uric acid concentrations among Koreans
Source: PLoS One. 2020 Apr 9;15(4):e0231336. doi: 10.1371/journal.pone.0231336 (PMC7145145; doi:10.1371/journal.pone.0231336)
Supplement: S1 Table — (PDF) [file pone.0231336.s004.pdf]

**S1 Table. The demographics of the study participants.**

| | GWAS stage | | Validation stage | | WGS* | |
|---|---|---|---|---|---|---|
| | Male | Female | Male | Female | Male | Female |
| Number of participants (%) | 1,902 (100) | 0 | 1,557 (53) | 1,355 (47) | 440 (55) | 357 (45) |
| Age (years) | 50 (45–55) | NA | 55 (48–63) | 53 (47–61) | 55 (49–63) | 54 (48–60) |
| Body mass index (kg/m$^2$) | 24.5 (22.9–26.4) | NA | 24.3 (22.3–26.0) | 22.6 (20.4–24.3) | 24.2 (22.2–25.9) | 22.4 (20.4–24.0) |
| Serum Uric Acid (mg/dL) | 6.4 (5.5–7.2) | NA | 6.2 (5.3–7.0) | 4.8 (4.1–5.4) | 6.3 (5.4–7.1) | 4.9 (4.1–5.5) |

Age, body mass index and serum uric acid were presented as median values with 25th–75th percentile values in parentheses.

*The entire 797 participants in the WGS-based association study were part of the validation stage.

WGS, whole genome sequencing; NA, not applicable
