# Supplementary material for: A population-specific low-frequency variant of SLC22A12 (p.W258*) explains nearby genome-wide association signals for serum uric acid concentrations among Koreans
Source: PLoS One. 2020 Apr 9;15(4):e0231336. doi: 10.1371/journal.pone.0231336 (PMC7145145; doi:10.1371/journal.pone.0231336)
Supplement: S2 Table — (PDF) [file pone.0231336.s005.pdf]

S2 Table. Linkage disequilibrium (LD) state (r^2) between single nucleotide polymorphisms (SNPs reaching a genome-wide significance on chromosome 11 65 Mb).

	rs184521656	rs117897057	rs78203666	rs185494956	rs117625825	rs140294098
rs140294098	0.66	0.95	0.95	0.98	0.98	1.00
rs117625825	0.68	0.95	0.98	1.00	1.00	
rs185494956	0.68	0.95	0.98	1.00		
rs78203666	0.71	0.97	1.00			
rs117897057	0.77	1.00				
rs184521656	1.00					
