# Supplementary material for: A population-specific low-frequency variant of SLC22A12 (p.W258*) explains nearby genome-wide association signals for serum uric acid concentrations among Koreans
Source: PLoS One. 2020 Apr 9;15(4):e0231336. doi: 10.1371/journal.pone.0231336 (PMC7145145; doi:10.1371/journal.pone.0231336)
Supplement: S3 Table — (PDF) [file pone.0231336.s006.pdf]

S3 Table. Selection of the tag SNPs.

Allele	Tag SNP	r^2
rs117897057	rs117625825	0.948
rs78203666	rs117625825	0.983
rs185494956	rs117625825	1
rs117625825	rs117625825	1
rs140294098	rs117625825	0.982
rs184521656	rs184521656	1
