# Supplementary material for: A population-specific low-frequency variant of SLC22A12 (p.W258*) explains nearby genome-wide association signals for serum uric acid concentrations among Koreans
Source: PLoS One. 2020 Apr 9;15(4):e0231336. doi: 10.1371/journal.pone.0231336 (PMC7145145; doi:10.1371/journal.pone.0231336)
Supplement: S4 Table — (PDF) [file pone.0231336.s007.pdf]

S4 Table. The association of rs184521656 and rs117625825 with serum uric acid concentration of males and females.

SNP	Chr	Position (bp)	Minor allele	Major allele	Male				Female			
					Number of participants	MAF (%)	Beta (SE)	*P*	Number of participants	MAF (%)	Beta (SE)	*P*
rs184521656	11	65161450	T	C	1470	1.5	-0.94 (0.19)	8.6E-07	1221	1.0	-1.01 (0.21)	1.2E-06
rs117625825	11	65765725	A	G	1469	1.6	-1.02 (0.18)	3.7E-08	1230	1.3	-0.54 (0.18)	2.3E-03

Chr, chromosome; MAF, minor allele frequency; SE, standard error
