# Supplementary material for: A population-specific low-frequency variant of SLC22A12 (p.W258*) explains nearby genome-wide association signals for serum uric acid concentrations among Koreans
Source: PLoS One. 2020 Apr 9;15(4):e0231336. doi: 10.1371/journal.pone.0231336 (PMC7145145; doi:10.1371/journal.pone.0231336)
Supplement: S5 Table — (PDF) [file pone.0231336.s008.pdf]

S5 Table. Association analysis of rs184521656, rs117625825 and rs549461 using whole genome sequencing (WGS) data before and after adjustment for rs121907892.

SNP	Chr	Position (bp)	Nearest genes	Minor allele	Major allele	LD (r^2) with rs121907892	WGS (n=797)			
							MAF (%)	Beta (SE)	*P*	*P* adjusted for rs121907892
rs184521656	11	65161450	*FRMD8* (intron)	T	C	0.32	1.5	-0.83 (0.26)	1.2E-03	4.5E-01
rs117625825	11	65765725	*EIF1AD* (3' UTR)	A	G	0.25	1.5	-0.74 (0.25)	2.9E-03	8.9E-01
rs549461	11	64423032	*SLC22A12-NRXN2* (intron)	A	G	0.06	18.8	-0.17 (0.08)	2.5E-02	5.0E-01

Chr, chromosome; WGS, whole genome sequencing; MAF, minor allele frequency; SE, standard error
