# Supplementary material for: A population-specific low-frequency variant of SLC22A12 (p.W258*) explains nearby genome-wide association signals for serum uric acid concentrations among Koreans
Source: PLoS One. 2020 Apr 9;15(4):e0231336. doi: 10.1371/journal.pone.0231336 (PMC7145145; doi:10.1371/journal.pone.0231336)
Supplement: S6 Table — (PDF) [file pone.0231336.s009.pdf]

S6 Table. Serum uric acid concentrations according to the rs121907892 genotype in the study population.

	Total		Male		Female	
Genotype	p.W258* heterozygote	Wild type	p.W258* heterozygote	Wild type	p.W258* heterozygote	Wild type
Number of Subjects	130	4,578	106	3,327	24	1,251
Mean (mg/dL)	4.03	5.95	4.21	6.38	3.25	4.81
SD	1.08	1.39	1.00	1.26	1.11	1.01

SD, standard deviation
