# Supplementary material for: A population-specific low-frequency variant of SLC22A12 (p.W258*) explains nearby genome-wide association signals for serum uric acid concentrations among Koreans
Source: PLoS One. 2020 Apr 9;15(4):e0231336. doi: 10.1371/journal.pone.0231336 (PMC7145145; doi:10.1371/journal.pone.0231336)
Supplement: S7 Table — (PDF) [file pone.0231336.s010.pdf]

S7 Table. Serum uric acid concentration of subjects with low-frequency variants of *SLC22A12*.

	Wid type	rs121907896 (R90H)	rs773677616 (R477H)	rs765990518 (Q382L)	Splicing (c.661+1G>A)
Number of subjects	729	9	2	1	1
Mean SUA (mg/dL) (Range)	5.7 (1.9 – 12.0)	3.9 (2.7 – 5.7)	3.5 (3.1 – 3.9)	2.9	4.2
