# Supplementary material for: A population-specific low-frequency variant of SLC22A12 (p.W258*) explains nearby genome-wide association signals for serum uric acid concentrations among Koreans
Source: PLoS One. 2020 Apr 9;15(4):e0231336. doi: 10.1371/journal.pone.0231336 (PMC7145145; doi:10.1371/journal.pone.0231336)
Supplement: S8 Table — (PDF) [file pone.0231336.s011.pdf]

S8 Table. Probes used for Taqman genotyping.

Assay Name	Product Type	Reporter Dye 1/2	Context Sequence [VIC/FAM]
RS184521656	Custom TaqMan® SNP Genotyping Assays, Human, MED	VIC/FAM	TGGCCAGGGCCTGGGCCAAT**[C/T]**GGGAGAGGGGAGGGCTAAGC
RS117625825	Custom TaqMan® SNP Genotyping Assays, Human, MED	VIC/FAM	GAAGGCCCAGAGCACGGCCT**[A/G]**GCACTTTCTAAGTGCCCAAT
RS121907892	TaqMan® SNP Genotyping Assays, Human, MED	VIC/FAM	GGCCTACGGTGTGCGGGACTG**[A/G]**ACACTGCTGCAGCTGGTGGTC
